# Transcription of HIV-1 at sites of intact latent provirus integration

**DOI:** 10.1084/jem.20240391

**Published:** 2024-08-14

**Authors:** Ana Rafaela Teixeira, Cintia Bittar, Gabriela S. Silva Santos, Thiago Y. Oliveira, Amy S. Huang, Noemi Linden, Isabella A.T.M. Ferreira, Tetyana Murdza, Frauke Muecksch, R. Brad Jones, Marina Caskey, Mila Jankovic, Michel C. Nussenzweig

**Affiliations:** 1 Laboratory of Molecular Immunology, The Rockefeller University, New York, NY, USA; 2 Tessera Therapeutics, Somerville, MA, USA; 3 Division of Infectious Diseases, Department of Medicine, Weill Cornell Medicine, New York, NY, USA; 4 Department of Infectious Diseases, Medical Faculty Heidelberg, Virology, Center for Integrative Infectious Disease Research (CIID), Heidelberg University, Heidelberg, Germany; 5 Department of Infectious Diseases, Virology, Chica and Heinz Schaller (CHS) Research Group, University Hospital Heidelberg, Heidelberg, Germany; 6 Howard Hughes Medical Institute, Chevy Chase, MD, USA

## Abstract

HIV-1 antiretroviral therapy is highly effective but fails to eliminate a reservoir of latent proviruses, leading to a requirement for life-long treatment. How the site of integration of authentic intact latent proviruses might impact their own or neighboring gene expression or reservoir dynamics is poorly understood. Here, we report on proviral and neighboring gene transcription at sites of intact latent HIV-1 integration in cultured T cells obtained directly from people living with HIV, as well as engineered primary T cells and cell lines. Proviral gene expression was correlated to the level of endogenous gene expression under resting but not activated conditions. Notably, latent proviral promoters were 100–10,000× less active than in productively infected cells and had little or no measurable impact on neighboring gene expression under resting or activated conditions. Thus, the site of integration has a dominant effect on the transcriptional activity of intact HIV-1 proviruses in the latent reservoir, thereby influencing cytopathic effects and proviral immune evasion.

## Teaser

This study uses reporter cells engineered with a minimal expression construct and cells cultured directly from the reservoir of HIV-1 infected individuals to show that proviral transcription in latently infected cells is predominantly dependent on the site of proviral integration.

## Introduction

HIV-1 proviral integration into the genome of CD4^+^ T cells typically leads to the production of up to 10^4^ virions per cell and results in cell death by apoptosis (Wei et al., 1995; Perelson et al., 1996). However, in a small number of instances, the integrated provirus remains latent but can be reactivated in vitro or in vivo upon treatment interruption (Pace et al., 2011; Cohn et al., 2020; Liu et al., 2020; Dufour et al., 2021; Siliciano and Siliciano, 2022). Antiretroviral therapy (ART) prevents the spread of productive infection and disease progression but does not eliminate a reservoir of infected cells that carry intact latent proviruses (Wong et al., 1997; Chun et al., 1998; Finzi et al., 1999). This reservoir represents the primary barrier to HIV-1 cure (Li et al., 2016; Margolis and Archin, 2017).

Obtaining a detailed understanding of the reservoir has been challenging because the cells that carry intact latent proviruses are rare and there are no definitive cell surface markers that can be used to isolate them (Ho et al., 2013; Crooks et al., 2015; Bachmann et al., 2019; Darcis et al., 2019; Cohn et al., 2020; Peluso et al., 2020; Weymar et al., 2022; Sun et al., 2023; Wong et al., 2023). Nevertheless, there has been a great deal of progress in understanding the nature of the reservoir (Pace et al., 2011; Cohn et al., 2020; Liu et al., 2020; Dufour et al., 2021; Siliciano and Siliciano, 2022). Documented features of the reservoir include the following: (1) CD4^+^ T cells that carry intact latent proviruses are oligoclonal (Lorenzi et al., 2016; Simonetti et al., 2016; Bui et al., 2017; Hosmane et al., 2017; Cohn et al., 2018; Lu et al., 2018); (2) the clones of cells carrying intact latent proviruses are dynamic, and while the overall number of T cells carrying latent proviruses decreases over time, they also become more clonal (Cho et al., 2022); (3) intact proviruses found in large CD4^+^ T cell clones are the least likely to be reactivated (Lorenzi et al., 2016); and (4) some intact proviruses in the latent reservoir are transcriptionally active and others are not (Einkauf et al., 2022).

During initial infection, integrase favors proviral deposition in the introns of highly expressed genes associated with regions of accessible chromatin (Schröder et al., 2002; Han et al., 2004; Craigie and Bushman, 2012). These proviruses are the first to be eliminated due to their cytopathic effects. Over time on ART, and in elite controllers, intact proviruses are enriched in heterochromatin, non-genic regions, and in an opposite orientation to the host gene (Einkauf et al., 2019, 2022; Jiang et al., 2020; Huang et al., 2021). In addition to centromeric and satellite DNA, integration into zinc finger (ZNF) genes is enriched possibly because these sites are less permissive for provirus expression and subsequent negative selection (Jiang et al., 2020; Huang et al., 2021). Consistent with this idea, experiments in transformed cell lines using randomly integrated HIV-1 reporter proviruses suggest that transcriptional activity is dependent on the site of integration (Jordan et al., 2001; Lewinski et al., 2005; Sherrill-Mix et al., 2013; Chen et al., 2017; Battivelli et al., 2018; Collora and Ho, 2023). However, the relationship between the site of authentic latent proviral integration and transcriptional activity or its effect on neighboring genes is not known. Understanding how the reservoir of intact latent proviruses is regulated and how it impacts neighboring gene expression in primary CD4^+^ T cells is critical to understanding how the reservoir is maintained and how it might be eliminated.

To address these questions, we analyzed HIV-1 and global gene expression in Jurkat and primary T cells that carry HIV-1 reporters inserted precisely into sites of intact latent provirus integration and in authentic latent CD4^+^ T cells isolated directly from people living with HIV (PLWH).

## Results

### HIV-1 LTR reporter expression by flow cytometry

**Figure 1.**
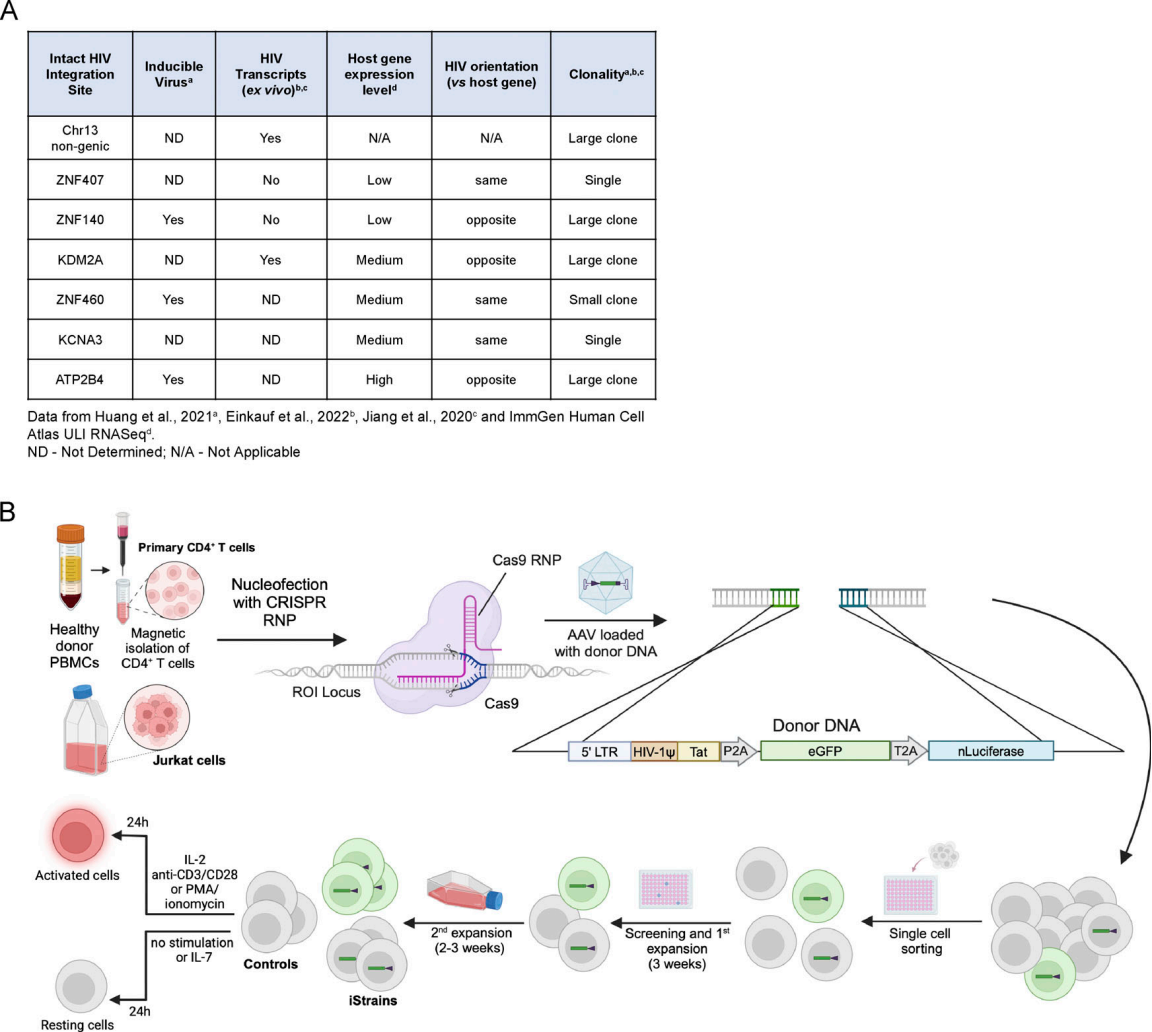
**Selected integration sites and production of reporter T cell clones. (A)** Table of selected integration sites. **(B)** Schematic representation of the methods used to produce reporter T cell lines (see Materials and methods for details). Created with https://BioRender.com.

To determine how intact proviruses alter gene expression or are influenced by their position in the host genome, we selected seven well-documented sites of latent HIV-1 integration obtained from PLWH, three of which had been demonstrated to be inducible in vitro (Fig. 1 A) (Jiang et al., 2020; Huang et al., 2021; Einkauf et al., 2022). The seven sites differed in their documented level of host gene expression, proviral orientation with respect to the neighboring gene, and whether HIV-1 transcripts were detected in primary cells (Fig. 1 A). Five of the seven sites were found among expanded clones and two in unique CD4^+^ T cells (Jiang et al., 2020; Huang et al., 2021; Einkauf et al., 2022). One was in a non-genic region of chr13 and the remainder were in introns of genes expressed at low (*ZNF407*, *ZNF140*), medium (*KDM2A*, *ZNF460*, *KCNA3*), and high (*ATP2B4*) levels (Fig. 1 A).

A reporter construct consisting of an HIV-1 long terminal repeat (LTR), transactivator of transcription (Tat), green fluorescent protein (eGFP), and nano Luciferase (nLuciferase) was inserted into the precise position of each of the seven intact latent proviruses using CRISPR ribonucleoproteins in Jurkat cells or primary CD4^+^ T cells. Integration was verified by PCR in expanded clones obtained from single cells (iStrains; Fig. 1 B). The amplified region encompassed the integration site and construct, and the integrity of both was confirmed by Sanger sequencing. Several independent clones were obtained for *ATP2B4* and *KCNA3* in Jurkat and CD4^+^ T cells and for *ZNF407* in Jurkat cells. We were unable to obtain an integration in *ZNF407* in primary CD4^+^ T cells. Control cells were transfected and cultured under the same conditions but did not contain a reporter.

**Figure 2.**
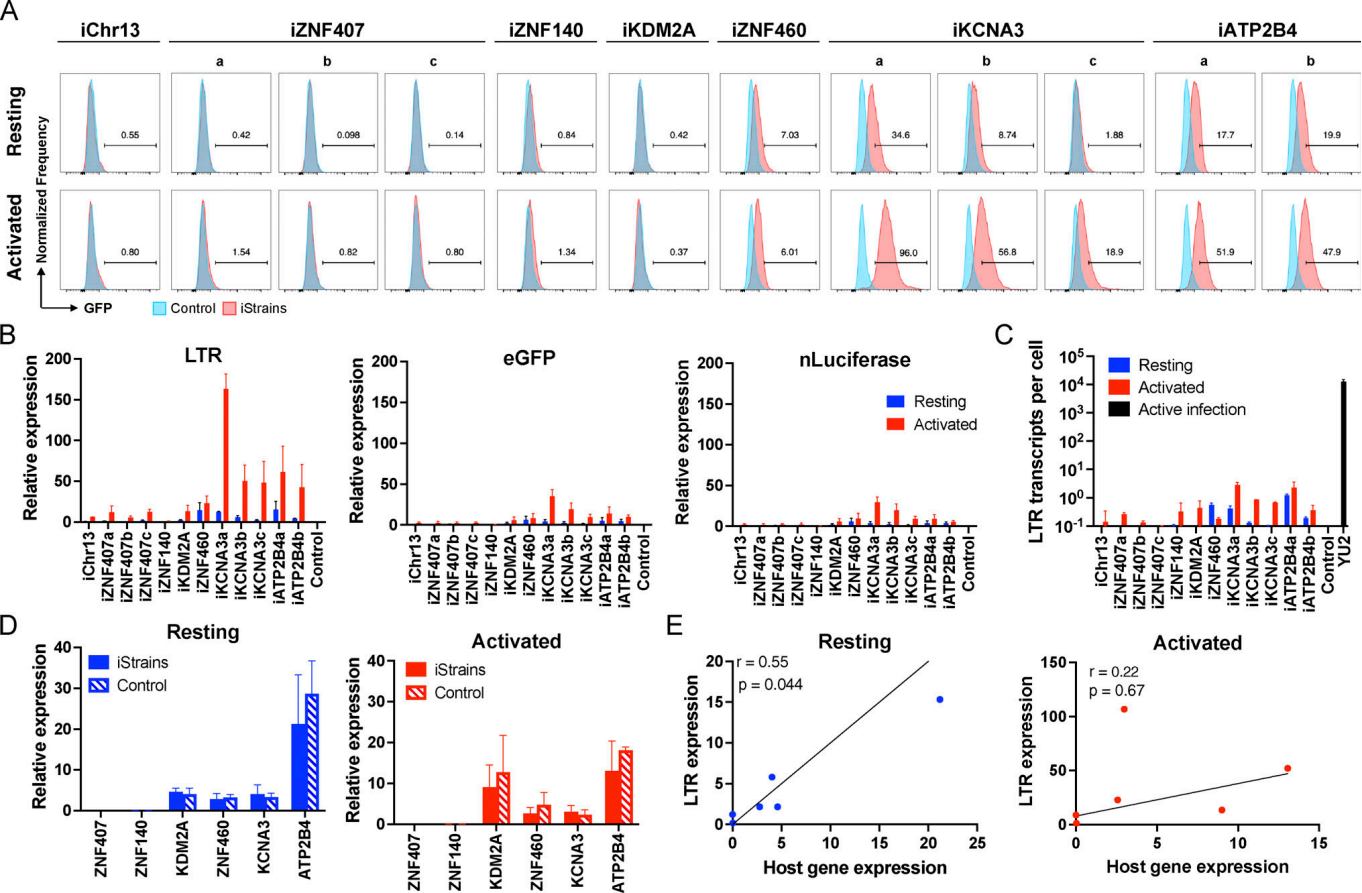
**Jurkat reporter lines. (A)** Histograms show GFP fluorescence (x axis) per normalized counts (y axis) for each integration site studied (iStrains, red) and control (blue), in both resting (upper panel) and PMA/ionomycin-activated (lower panel) conditions. **(B)** Graphs show relative LTR (left panel), eGFP (middle panel), and nLuciferase (right panel) expression assessed by qPCR under resting (blue) and PMA/ionomycin-activated (red) conditions. Bars represent the mean relative expression from two independent assays (biological replicates) ± SD. **(C)** LTR transcripts per cell (y axis), determined by qPCR, for each integration-positive clone and control, under resting (blue) and activated (red) conditions. CD4^+^ T cells infected with HIV-1_Y__U__2_ served as positive control (black bar). Bars represent the mean of two independent assays (biological replicates) ± SD. **(D)** Relative expression determined by qPCR for host genes neighboring their respective reporter proviruses (iStrains, full bars) and control (striped bars), under resting (left panel, blue), and PMA/ionomycin-activated (right panel, red) conditions. Bars represent the mean relative expression of two independent assays (biological replicates) ± SD. Expression of the host gene was averaged across multiple clones to the same reporter integration site. **(E)** Correlation between the relative expression of LTR (y axis) for each Jurkat clone and their respective host gene (x axis), averaged across multiple clones to the same reporter integration site, under resting (left panel, blue) and PMA/ionomycin-activated (right panel, red) conditions. Pearson’s correlation coefficients, r, and two-tailed P values were computed for each condition.

**Figure 3.**
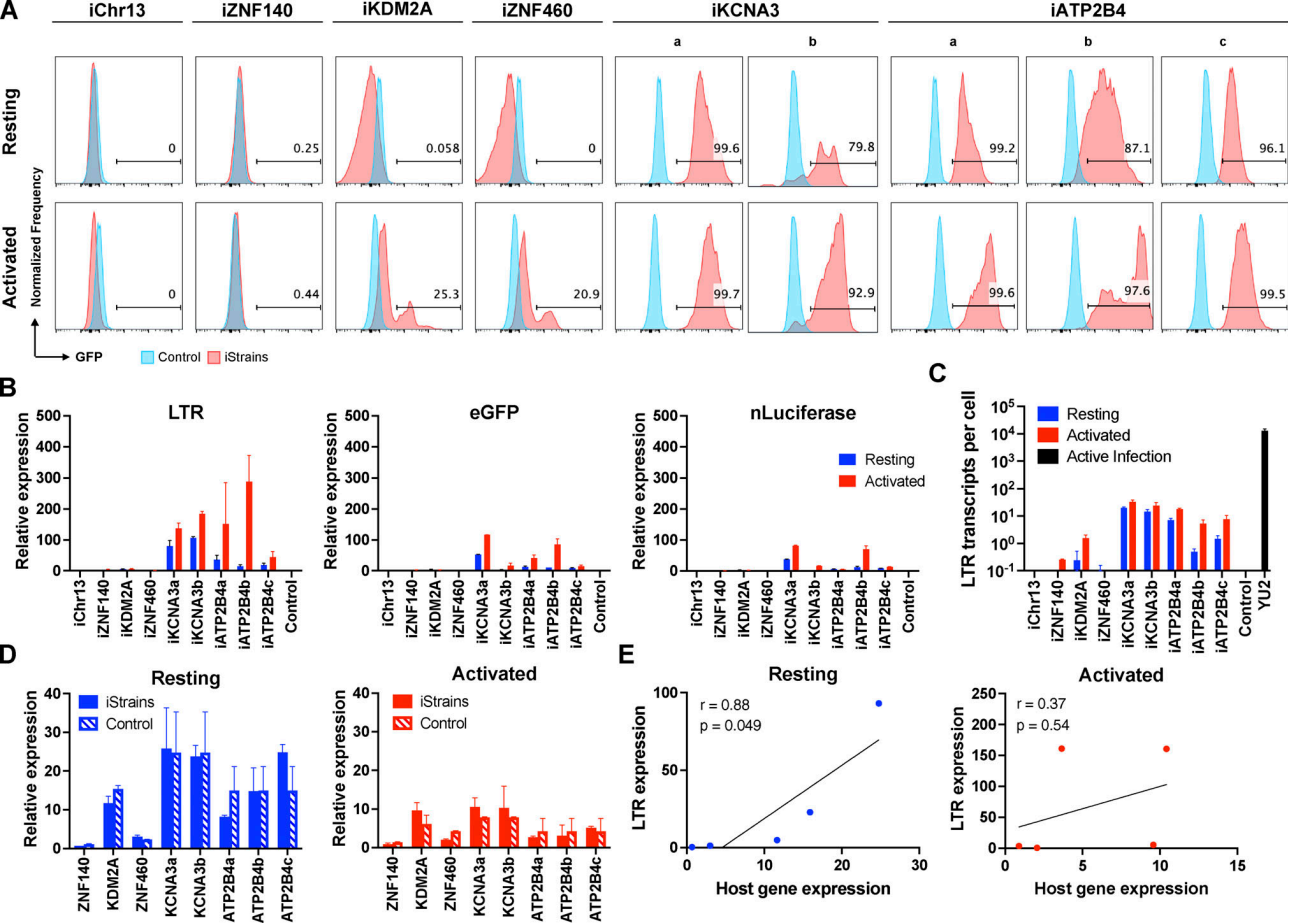
**Primary CD4** ^**+**^ **T cell reporter lines. (A)** Histograms show GFP fluorescence (x axis) per normalized counts (y axis) for each integration site studied (iStrains, red) and control (blue) in both resting (upper panel) and CD3/CD28-activated (lower panel) conditions. **(B)** Graphs show relative LTR (left panel), eGFP (middle panel), and nLuciferase (right panel) expression assessed by qPCR under resting (blue) and CD3/CD28-activated (red) conditions. Bars represent the mean relative expression from two independent assays (biological replicates) ± SD. **(C)** LTR transcripts per cell (y axis), determined by qPCR, for each integration-positive clone and control, under resting (blue) and CD3/CD28-activated (red) conditions. CD4^+^ T cells infected with HIV-1_Y__U__2_ served as positive control (black bar). Bars represent the mean of two independent assays (biological replicates) ± SD. **(D)** Relative expression determined by qPCR for host genes neighboring their respective reporter proviruses (iStrains, full bars) and control (striped bars) under resting (left panel, blue) and CD3/CD28-activated (right panel, red) conditions. Bars represent the mean relative expression of two independent assays (biological replicates) ± SD. **(E)** Correlation between the relative expression of LTR (y axis) for each primary T cell clone and their respective host gene (x axis), averaged across multiple clones to the same reporter integration site, under resting (left panel, blue) and activated (right panel, red) conditions. Pearson’s correlation coefficients, r, and two-tailed P values were computed for each condition.

**Figure S1.**
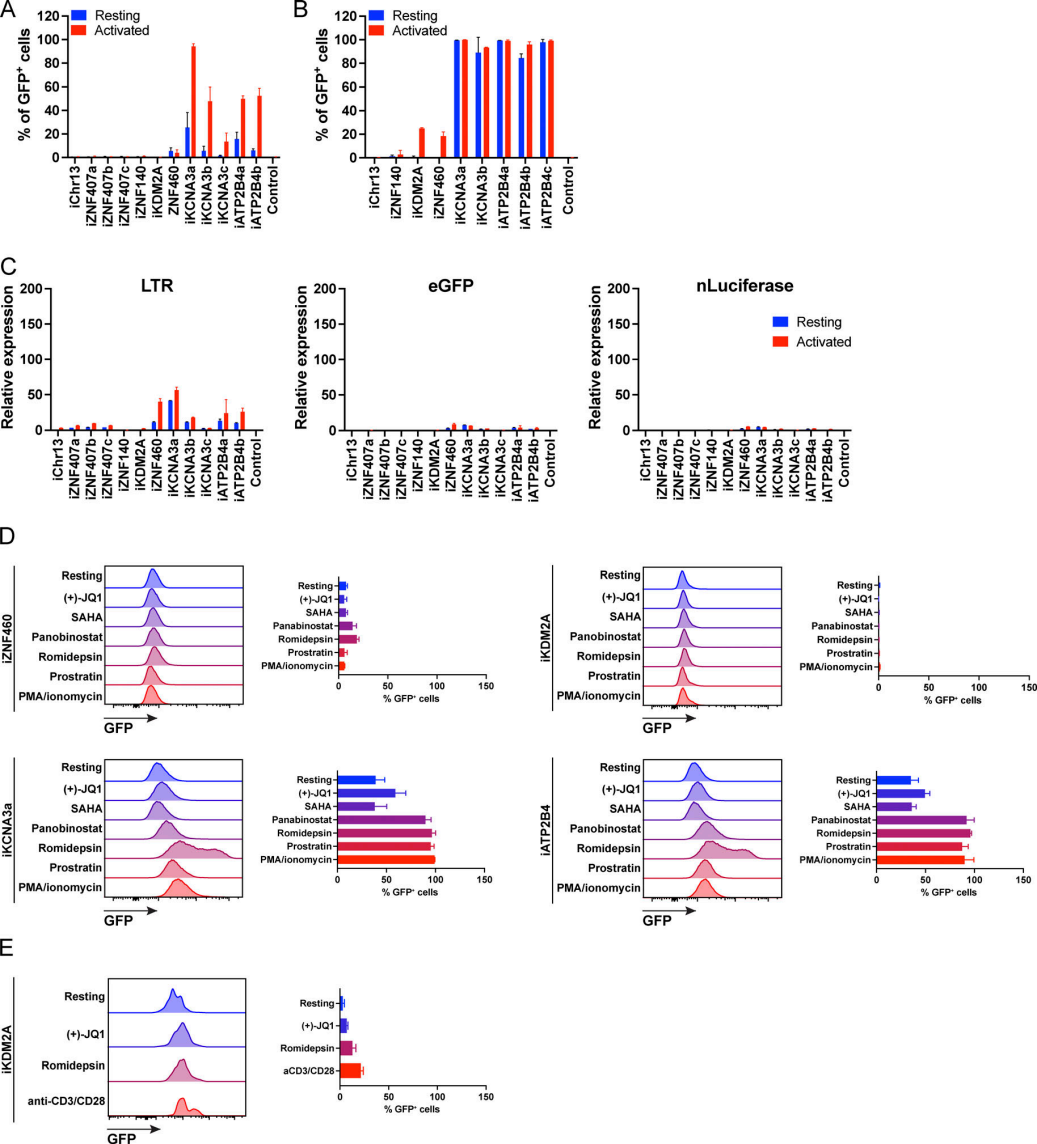
**GFP expression in reporter cell lines. (A and B)** Percentage of GFP^+^ cells as measured by flow cytometry for each integration-positive clone and control, under resting (blue) and PMA/ionomycin- and CD3/CD28-activated (red) conditions, respectively for Jurkat (A) and primary CD4^+^ T (B) cells. Bars represent the mean of two independent experiments (biological replicates) ± SD. **(C)** Graphs showing relative LTR (left panel), eGFP (middle panel), and nLuciferase (right panel) expression assessed by qPCR in Jurkat cell clones under resting (blue) and CD3/CD28-activated (red) conditions. Bars represent the mean relative expression from two independent assays (biological replicates) ± SD. **(D and E)** Representative histograms (left panels) show GFP fluorescence (x axis) per normalized counts (y axis) for Jurkat clones iZNF460, iKDM2A, iKCNA3a, and iATP2B4a (D), and primary CD4^+^ T cell clone iKDM2A (E), cultured for 24 h under resting conditions or in the presence of different LRAs or cell activation stimuli (PMA/ionomycin for Jurkat cells, anti-CD3/CD28 monoclonal antibodies for primary cells). Graphs (right panels) show the percentage of GFP^+^ cells measured by flow cytometry for the same conditions. Bars represent the mean of two independent experiments (biological replicates) ± SD.

As measured by flow cytometry, reporter expression varied depending on the integration site. There was no detectable expression by reporters integrated into *ZNF140* and *ZNF407,* or a non-genic region in chr13 in Jurkat cells or primary CD4^+^ T cells under resting conditions or after activation with phorbol 12-myristate 13-acetate (PMA) and ionomycin or anti-CD3 and -CD28 monoclonal antibodies, respectively (Fig. 2 A and Fig. 3 A). The reporters integrated into *ZNF460* and *KDM2A* were also silent in primary CD4^+^ T cells, but in contrast to the other three silent genes, they were expressed in a fraction of the cells upon activation (Fig. 3 A). Reporters integrated into *KCNA3* and *ATP2B4* from multiple independent clones were expressed under resting conditions, and expression was further enhanced by activation, as measured by an increase in the percentage of cells expressing GFP and in their mean fluorescence intensity (Fig. 2 A, Fig. 3 A, and Fig. S1, A and B). To determine whether reporter expression can be enhanced by latency reversal agents (LRAs), we treated Jurkat or primary T cells with LRAs and analyzed them by flow cytometry (Fig. S1, D and E). Jurkat clones expressing GFP in resting state, iZNF460, iKCNA3, and iATP2B4, treated with romidepsin, panobinostat, and prostratin showed enhanced expression of GFP to levels comparable to activation with PMA/ionomycin (Fig. S1 D). In contrast, the expression of the reporter integrated into the silent *KDM2A* gene was not altered by the LRAs tested (Fig. S1 D). In primary T cells carrying integration in *KDM2A*, romidepsin enhanced GFP expression significantly but to a lower level than anti-CD3 and -CD28 antibody–mediated activation (Fig. S1 E). Thus, LRA-induced expression is also integration site dependent.

### Proviral RNA expression

Transcription of intact HIV-1 proviruses in the latent reservoir has been documented by sensitive single-copy PCR assays (Einkauf et al., 2022). However, the assay involves multiple rounds of polymerase amplification and is not quantitative. Thus, the amount of transcription at the sites of intact latent proviral integration, how it compares to productive infection, and how it relates to the site of integration have not been examined.

We performed quantitative PCR (qPCR) assays to determine the level of transcription of integrated proviral reporters under resting and activated conditions in Jurkat and primary CD4^+^ T cell lines (Fig. 2 B, Fig. 3 B, and Fig. S1 C). Under resting conditions, reporter LTR expression was either very low at chr13, *ZNF407*, *ZNF140*, *KDM2A*, and *ZNF460* sites in Jurkat cell lines (Fig. 2 B) or undetectable in primary CD4^+^ T cells (Fig. 3 B). eGFP and nLuciferase were transcribed at lower levels than the LTR and only detectable in reporters integrated at *KCNA3* and *ATP2B4* in primary CD4^+^ T cells suggesting incomplete transcript elongation (Fig. 2 B and Fig. 3 B). Activation with PMA/ionomycin or anti-CD3/28 antibodies increased LTR mRNA expression to measurable levels at all sites of integration except *ZNF140* in Jurkat cells (Fig. 2 B and Fig. S1 C). In contrast, only reporters integrated into *KCNA3* and *ATP2B4* showed increased LTR expression in primary CD4^+^ T cells (Fig. 3 B). However, even after activation, the level of reporter LTR expression, quantified as the number of transcripts per cell, was 10^3^–10^4^ and 10^2^–10^3^-fold lower than HIV-1_YU2_ infected CD4^+^ T cells in Jurkat and primary CD4^+^ T cells, respectively (Fig. 2 C and Fig. 3 C). Thus, the results in Jurkat and primary CD4^+^ T cells are generally congruent, but reporter expression levels are lower in Jurkat cell lines.

### Host gene expression and chromatin architecture

**Figure S2.**
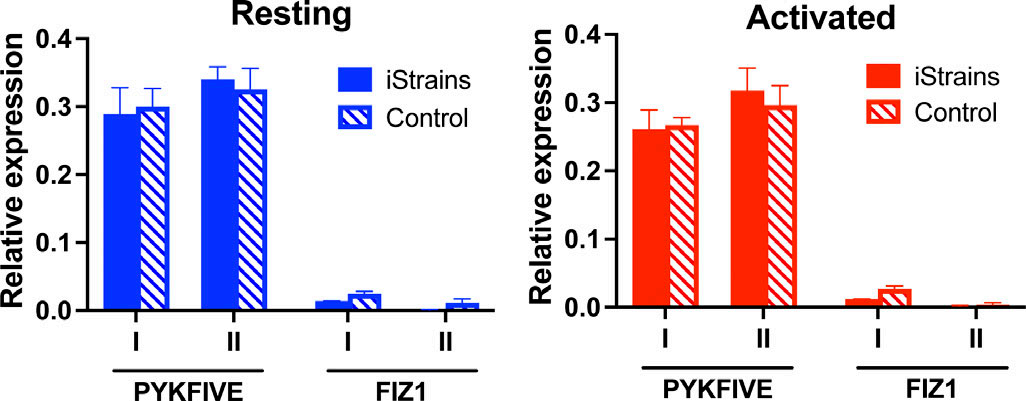
**Expression of host genes carrying reporter proviruses in Jurkat clones.** Relative expression of host genes *PYKFYVE* and *Fiz1* in Jurkat clones (full bars) and control (striped bars), under resting (blue) and activated (red) conditions. Bars represent the mean relative expression ± SD for each gene for three technical replicates, for two independent assays (biological replicates, I and II).

To determine how proviruses affect nearby host genes, we measured their expression by qPCR under resting and activated conditions. In all cases in Jurkat and primary CD4^+^ T cells, host gene mRNA expression was not significantly altered by reporter integration (Fig. 2 D and Fig. 3 D). Similar results were obtained using a different reporter that included two LTRs (Fig. S2). Under resting conditions, reporter LTR expression in Jurkat and primary CD4^+^ T cells was directly but not precisely proportional to the level of expression of the host gene (Fig. 2 E and Fig. 3 E). For example, in *ZNF140* and *ZNF460*, reporter LTRs are expressed at very low levels in both Jurkat and primary CD4^+^ T cells. However, *KDM2A* is expressed at relatively high levels in primary CD4^+^ T cells but the reporter is silent (Fig. 3 E). Notably, there was no significant correlation between the integration site and proviral transcription after activation, suggesting that under these conditions, regulation is no longer dominated by the host gene.

**Figure 4.**
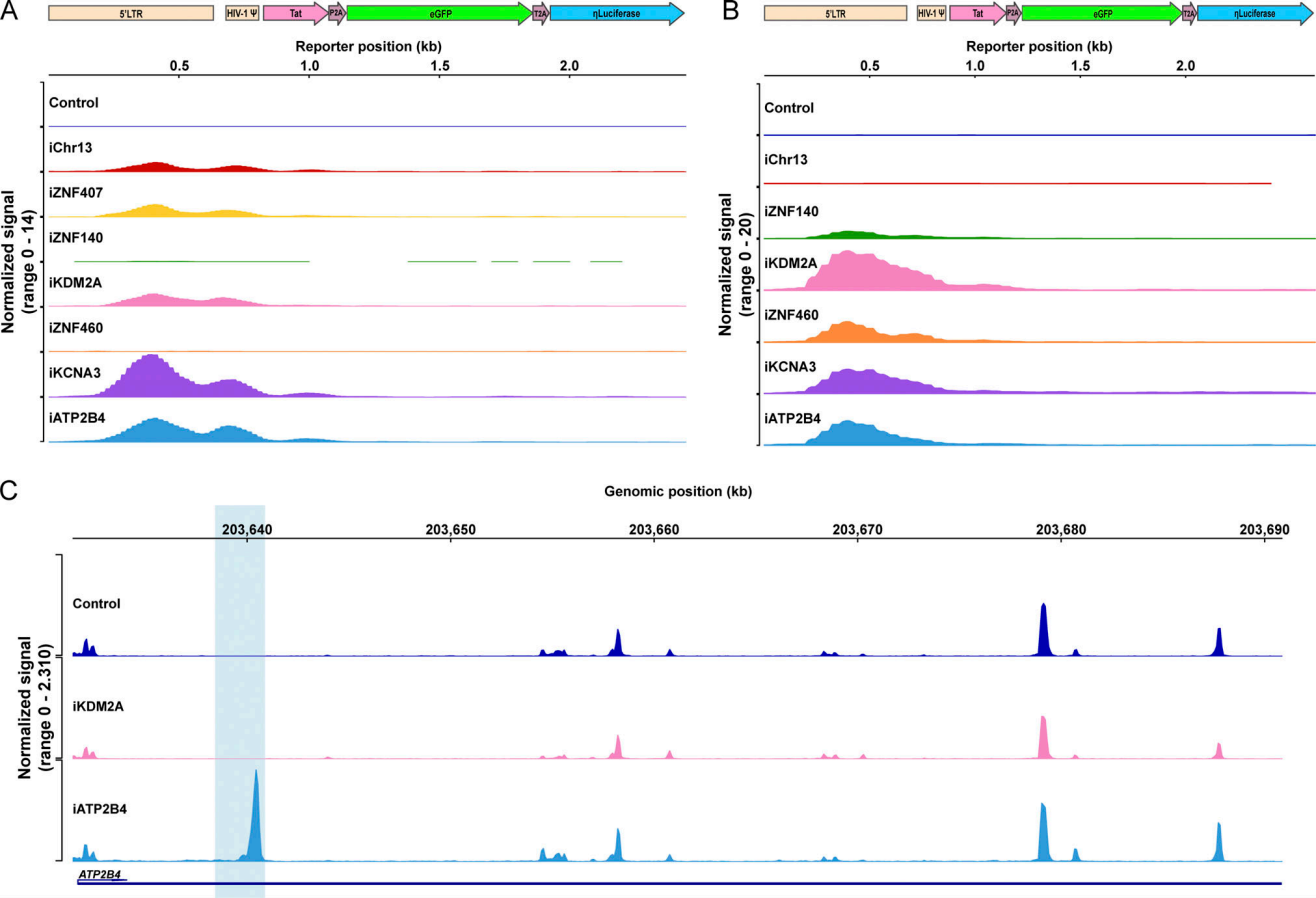
**Chromatin accessibility. (A and B)** Chromatin accessibility in Jurkat (A) and primary CD4^+^ T cell clones (B) as measured by ATAC-seq for the reporter construct at each integration site and control under resting conditions. **(C)** Graph shows ATAC-seq for *ATP2B4* encompassing the reporter integration site in control and clones that carry the reporter in *KDM2A*, *ATP2B4*. Blue shading indicates the site of reporter integration. Graphs were generated by averaging the normalized reads from three technical replicates for each clone.

**Figure S3.**
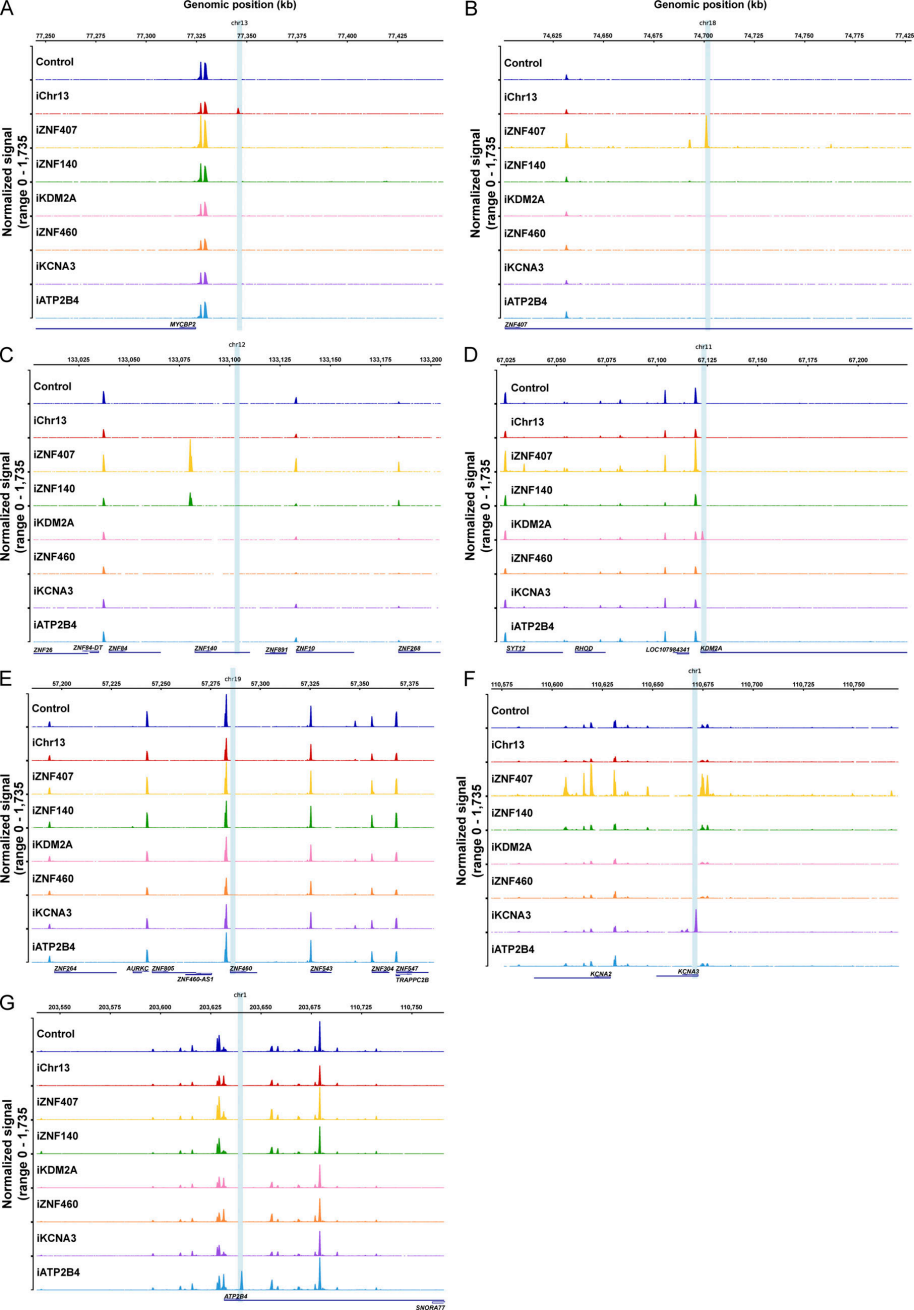
**Chromatin accessibility around reporter construct integration site in Jurkat cell clones. (A–G)** Chromatin accessibility measured by ATAC-seq in a 200,000 kb window of the genome around each of the integration sites for all Jurkat clones: chr13 (A), chr18 (*ZNF407*, B), chr12 (*ZNF140*, C), chr11 (*KDM2A*, D), chr19 (*ZNF460*, E), chr1 (*KCNA3*, F), and chr1 (*ATP2B4*, G). Graphs were generated by averaging the normalized reads from three technical replicates for each clone.

**Figure S4.**
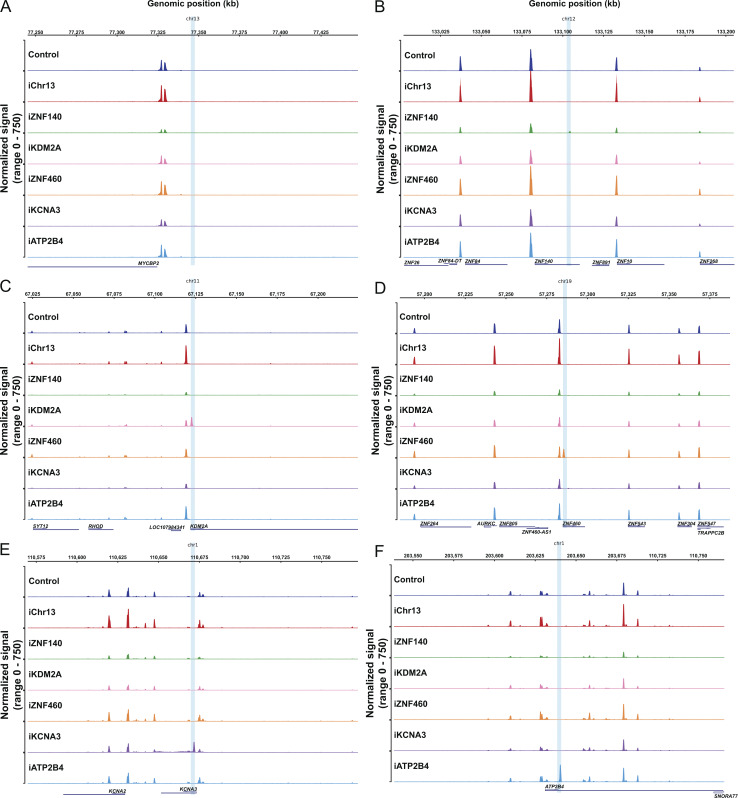
**Chromatin accessibility around reporter construct integration site in primary CD4** ^**+**^ **T cell clones. (A–F)** Chromatin accessibility measured by ATAC-seq in a 200,000 kb window of the genome around each of the integration sites for all primary CD4^+^ T cell clones: chr13 (A), chr12 (*ZNF140*, B), chr11 (*KDM2A*, C), chr19 (*ZNF460*, D), chr1 (*KCNA3*, E), and chr1 (*ATP2B4*, F). Graphs were generated by averaging the normalized reads from three technical replicates for each clone.

To determine how proviral integration might alter genome accessibility in or near the site of integration, we performed an assay for transposase-accessible chromatin with sequencing (ATAC-seq) on all Jurkat and primary CD4^+^ T cell lines. The reporter LTR was accessible in all cell lines except in *ZNF140* and, surprisingly, *ZNF460* in Jurkat cells and the non-genic region in chr13 in primary CD4^+^ T cells (Fig. 4, A and B). Thus, even in the absence of measurable transcription, the reporter remained accessible in most sites of latent integration. Notably, except for *KCNA3* in primary CD4^+^ T cells, the presence of the reporter did not alter neighboring genome chromatin accessibility irrespective of proviral transcription under resting or activated conditions (Fig. 4 C; and Figs. S3 and S4). We conclude that HIV-1 LTR reporter integration at authentic latent sites in Jurkat or primary CD4^+^ T cells does not alter local gene expression and only occasionally impacts local chromatin accessibility.

### Intact latent HIV-1 proviruses

**Figure 5.**
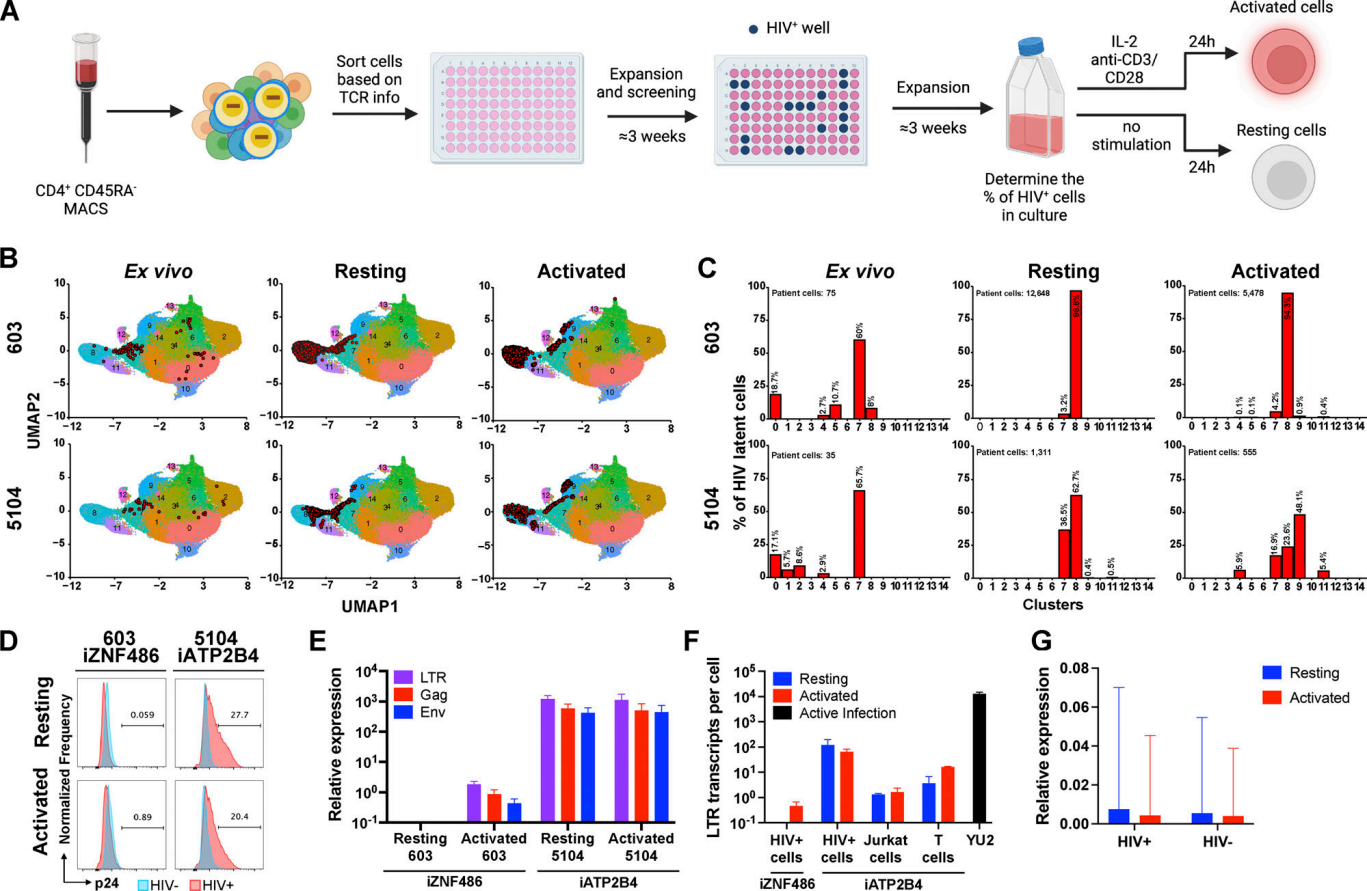
**HIV-infected cells from PLWH. (A)** Schematic representation of the methods used to grow out HIV-1–infected cells from ART-suppressed individuals (Weymar et al., 2022). Created with https://BioRender.com. **(B and C)** UMAP of 10X single-cell gene expression data showing the position of the cells expressing the latent clones’ specific TCR (red dots) (B) and the fraction of latent cells in each cluster (C), for participants 603 (upper panels) and 5104 (lower panels) from ex vivo cells (left panels, data from Weymar et al. [2022]), and cultured cells under resting (middle panels) and activated (right panels) conditions. **(D)** Histograms show HIV-1 Gag p24 expression in HIV^+^ cells (red) and non-infected cells (blue) of cultures derived from 603 to 5104, under resting (upper panel) and activated (lower panel) conditions. **(E)** Relative expression of LTR (purple bars), *gag* (red bars), and *env* (blue bars) by qPCR for 603 and 5104 HIV^+^ cells under resting and activated conditions. Bars represent the mean relative expression of two independent assays (biological replicates) ± SD. **(F)** LTR transcripts per cell determined by qPCR, in cells from 603 to 5104 and in Jurkat and primary T cells reporter lines with proviruses integrated into *ATP2B4*, under resting (blue) and activated (red) conditions and HIV-1_YU2_ controls. Bars represent the mean of two independent experiments (biological replicates) ± SD. **(G)** Relative expression determined by 10X Genomics single-cell mRNA sequencing of host gene neighboring HIV-1 proviral integration (*ZNF486*) in HIV-infected (HIV^+^) and non-infected (HIV^−^) cells from the same cell population under resting (blue bars) and activated (red bars) conditions. Bars represent the mean of the respective population from one assay ± standard deviation representing population variance.

CD4^+^ T cells carrying intact latent HIV-1 proviruses are rare and express no singular cell surface marker that distinguishes them from T cells other than their unique T cell receptor (TCR) (Cohn et al., 2018; Collora et al., 2022; Einkauf et al., 2022; Weymar et al., 2022; Sun et al., 2023; Wu et al., 2023). To examine HIV-1 expression in authentic latent reservoir cells, we enriched CD4^+^ T cells carrying replication-competent latent proviruses by means of their specific TCRs (Weymar et al., 2022): #603 and #5104 with HIV-1 integrated into *ZNF486* and human satellite 3 (HSat3) DNA in Chr22. The sorted cells were then expanded under limiting dilution conditions in the presence of irradiated feeder cells and antiretroviral drugs (ARVs). The presence and frequencies of the infected clones in the expanded cells lines were verified by PCR assays for their specific TCRs and HIV-1 sequences (Fig. 5 A). Cell lines were obtained in which at least half of the cells represented the T cell clones of interest with intact proviruses integrated into *ZNF486* and Chr22 HSat3.

Primary CD4^+^ T cells carrying intact latent proviruses isolated directly from individuals 5104 and 603 are enriched in T cell populations with specific transcriptional profiles (Weymar et al., 2022). To determine the transcriptional profile of cultured lines of CD4^+^ T cells carrying integrated HIV-1 proviruses in *ZNF486* and Chr22 HSat3, we performed single-cell mRNA sequencing experiments using the 10X Genomics platform. Specific TCR expression was used to identify the latent cells and map them onto our previous data set using a cut-off of 95% confidence (Fig. 5, B and C and Materials and methods) (Weymar et al., 2022). Cultured and ex vivo cells containing latent HIV-1 proviruses showed closely related transcriptional profiles (Fig. 5, B and C).

The enriched populations of resting (3 wk after activation) and activated (24 h after anti-CD3 and -CD28 monoclonal antibody stimulation) CD4^+^ T cells carrying the 5104 and 603 HIV-1 proviruses were initially examined for p24 expression by flow cytometry (Fig. 5 D). The HIV-1 provirus integrated into Chr22 HSat3 showed expression under both resting and activated conditions (Fig. 5 D). In contrast, the provirus integrated into *ZNF486*, a gene expressed at only low levels, failed to show detectable p24 expression under resting or activated conditions (Fig. 5 D).

**Figure S5.**
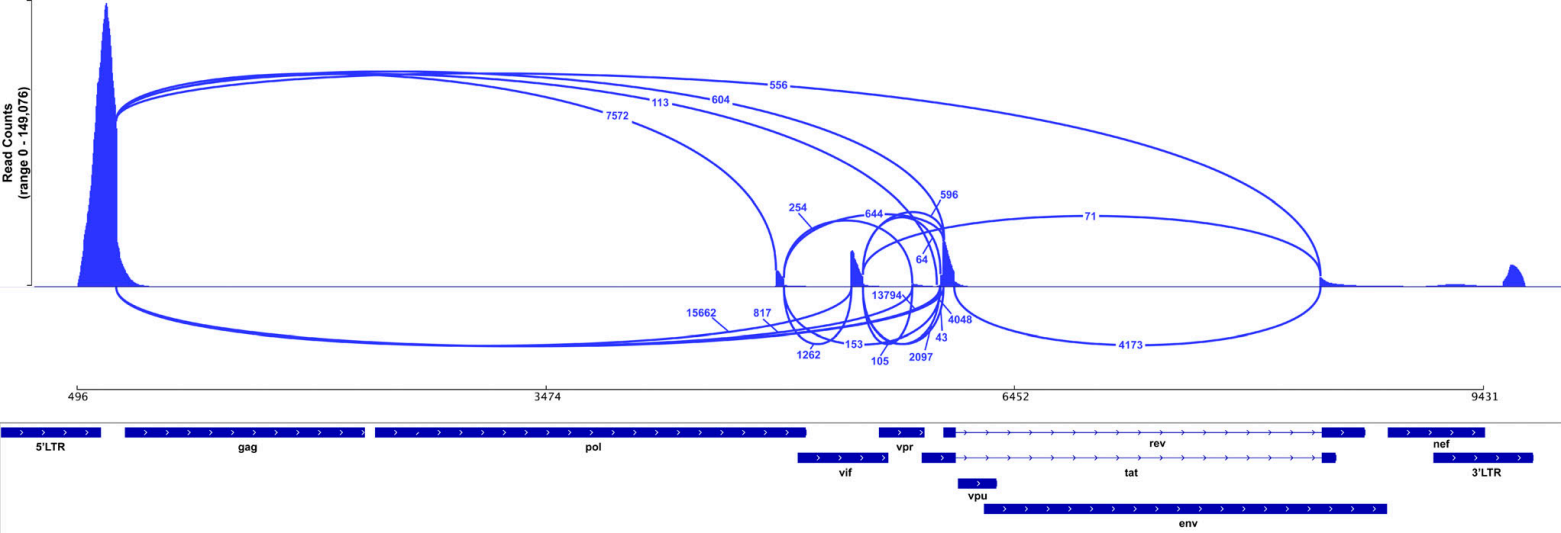
**HIV-1 RNA splice variants.** Sashimi plot showing scRNA-seq reads of 10X single-cell gene-expression data mapping to the HIV genome (bottom), in infected cells of participant 5104 under resting conditions. Splice junctions are represented as arcs connecting exons and the histogram represents the read coverage at each junction. The number of reads mapping to each junction is indicated by the numbers associated with each arc.

HIV-1 LTR, *gag*, and *env* mRNA expression were measured by qPCR corrected for the fraction of infected cells in the culture (Fig. 5 E and Materials and methods). The HIV-1 provirus integrated into Chr22 HSat3 showed similar levels of expression of LTR, *gag*, and *env* under resting and activated conditions. In addition, we found multiple spliced transcripts associated with productive infection in single-cell transcriptome analysis (Fig. S5). In contrast, the HIV-1 provirus integrated into *ZNF486* only showed LTR, *gag*, and *env* transcripts after activation (Fig. 5 E). When compared to productive infection with HIV-1_YU2_, the proviruses in Chr22 HSat3 and *ZNF486* were expressed at >100 and >27,000-fold lower levels, respectively (Fig. 5 F). When compared with reporter proviruses integrated into *ATP2B4,* the level of LTR expression obtained from cultured CD4^+^ T cells carrying latent HIV-1 was 10- and 100-fold higher than in primary CD4^+^ T cells and Jurkat cells, respectively. We conclude that authentic latent HIV-1 proviruses integrated at different sites in the genome of CD4^+^ T cells show different levels of expression and ability to respond to activation. In addition, proviruses integrated at latent sites are expressed at far lower levels than in productive infection.

To determine whether the latent HIV-1 proviruses in the cultured cells altered neighboring host gene transcription (*ZNF486*), their expression was compared with non-infected cells from the same culture in the 10X Genomics data set. We found no significant difference in the expression of the gene neighboring the integrated HIV-1 provirus between infected and non-infected cells irrespective of activation (Fig. 5 G).

## Discussion

HIV-1 infection typically results in the death of the infected cell. However, CD4^+^ T cells harboring intact chromosomally integrated HIV-1 proviruses can persist for years in individuals who are virally suppressed on ART (Dufour et al., 2021; Lichterfeld et al., 2022; Siliciano and Siliciano, 2022). The provirus is silent in some of these latent cells, which may explain why they persist, but intact proviral transcription has been detected using sensitive but non-quantitative PCR methods (Einkauf et al., 2022). We have examined HIV-1 proviral transcription at sites of authentic intact latent proviral integration in Jurkat cell lines, primary CD4^+^ T cells, and CD4^+^ T cell lines derived from PLWH. The data reveal that in resting, but not activated conditions, the transcriptional activity at the site of intact proviral integration is correlated to the level of proviral gene expression. Notably, the intact proviruses studied had little or no detectable impact on neighboring gene expression.

The reservoir of CD4^+^ T cells that harbor latent proviruses is both heterogeneous and dynamic. It is composed of clones of CD4^+^ T cells that expand and contract in a manner that is in part dependent on antigenic stimulation and trophic cytokines required for CD4^+^ T cell longevity (Demoustier et al., 2002; Douek et al., 2002; Chomont et al., 2009; Saleh et al., 2011; Jones et al., 2012; Cohn et al., 2015; Lorenzi et al., 2016; Bui et al., 2017; Hosmane et al., 2017; Lee et al., 2017; Pinzone et al., 2019; De Scheerder et al., 2019; Antar et al., 2020; Mendoza et al., 2020; Simonetti et al., 2021; Cho et al., 2022). Over time, the measurable reservoir contracts with an initial half-life of ∼4 years and becomes more clonal (Siliciano et al., 2003; Crooks et al., 2015; Bachmann et al., 2019; Peluso et al., 2020; Cho et al., 2022). Larger clones of CD4^+^ T cells harboring intact proviruses appear to be more difficult to reactivate (Lorenzi et al., 2016), and their proviruses are preferentially found in transcriptionally inactive parts of the genome (Einkauf et al., 2019; Huang et al., 2021). Sensitive PCR-based methods indicate that this shift in the reservoir is associated with retroviral transcription with preferential survival of silent clones (Einkauf et al., 2022). Thus, the transcriptional status of the intact provirus appears to be an essential element in determining its longevity. Our experiments indicate that there is a great deal of variability in the level of latent proviral expression that tracks with integration site and cell type. How the site of integration of intact latent proviruses or its transcription might alter latent cell longevity is not known, but the mRNA or protein products of the virus could have a direct effect on the cell or indirectly engage host immunity.

We found little effect on transcription of neighboring genes by reporter proviruses or HIV-1 integrated at nine sites of intact latent proviral integration in Jurkat cell lines, primary CD4^+^ T cells, or CD4^+^ T cells obtained from PLWH and grown in culture. Initially, intact latent proviruses are preferentially found in introns of genic regions in the opposite transcriptional orientation to the transcriptional start site (Einkauf et al., 2019; Huang et al., 2021). Over time, in chronically infected individuals under ART and elite controllers, intact latent proviruses accumulate in ZNF genes and non-genic regions (Einkauf et al., 2019; Jiang et al., 2020; Huang et al., 2021). Our collection of integration sites mirrors this selection, and therefore the observation that intact latent proviruses have little measurable effect on the neighboring genome may be biased by the origins of the sample set. Nevertheless, the integrations examined include unique proviruses found only once and others found in expanded clones.

In contrast to the absence of proviral influence on neighboring genes, the transcriptional status of the proviruses was directly correlated to that of the genes at integration sites. Notably, the correlation between the proximal host gene expression and LTR transcription was lost upon T cell activation. In some instances, proviral transcription was increased even as proximal host gene expression was downregulated. These results are consistent with a baseline Tat-independent phase during which basal expression depends mostly on host factors and a second activation phase in which a Tat-dependent positive feedback loop promotes proviral transcription (Jordan et al., 2001). Consistent with this idea, proviruses integrated in highly repressed genomic regions, i.e., *ZNF140* or in non-genic regions, that do not express Tat, remained silent after T cell stimulation. Our findings are consistent with observations made with randomly integrated reporter proviruses in cell lines that the level of HIV expression depends on the DNA integration site (Jordan et al., 2001, 2003; Lewinski et al., 2005; Skupsky et al., 2010). However, our sample size is limited, and a larger and more diverse group of authentic latent integration sites is necessary to understand the overall influence of integration sites on proviral expression precisely. Moreover, our reporters are missing components of the provirus that can impact the host cell and promote proviral survival and transcription (Malim and Emerman, 2008; Cesana et al., 2017; Pinzone et al., 2019; Yeh et al., 2020). Their absence may in part explain why transcription from the indicator lines is lower than the authentic latently infected cell lines carrying an intact provirus in the same genomic location.

Notably, we found somewhat variable levels of expression between clones carrying the same integration. In addition, only a fraction of CD4^+^ T cells expressed the reporters integrated in *KDM2A* and *ZNF460* after activation. Similarly, only a fraction of the latently infected cells obtained from PLWH expressed p24. Our observations are consistent with the finding that HIV-1 transcription is inherently stochastic (Ne et al., 2018; Mori and Valente, 2020), which may result in varying levels of gene expression in single cells within a clonal population. This effect may be further compounded by the stochasticity of host gene transcription in the clones (Raj and Oudenaarden, 2008). This variability may in part explain why activation of proviruses in vitro requires multiple rounds of stimulation (Hosmane et al., 2017).

We studied two cell lines derived directly from latently infected cells obtained from PLWH. Proviral transcription was detected in cells where HIV-1 was integrated in Chr22 HSat3, but not in cells carrying a provirus integrated in *ZNF486*, a gene expressed at low levels. In all cases, the levels of HIV-1 transcription from sites of intact latent integration were at least two orders of magnitude below the levels found in productively infected cells. Although insufficient to kill all the infected cells, the level of proviral transcription from the Chr22 HSat3 integration was sufficient to produce an infectious virus (data not shown). Thus, latent CD4^+^ T cells carrying an HIV-1 provirus integrated into Chr22 HSat3 are also likely to produce enough viral protein to be visible to the immune system.

In conclusion, the direct relationship between integration site and transcriptional regulation provides a road map for understanding how individual proviruses in the reservoir are selected against and how they contribute to viral rebound.

## Materials and methods

### Participant material and cell lines

Jurkat cells were procured from ATCC (clone E6-1, TIB-152). Primary CD4^+^ T cells were isolated from frozen peripheral blood mononuclear cells (PBMCs) of a healthy individual, while latent HIV^+^ cells were isolated as described below from frozen PBMCs of participants of a previous study. Study participants were recruited at the Rockefeller University Hospital and gave informed written consent before participation in the study. The study protocols and procedures (Rockefeller University IRB–approved Protocols MCA-0866/TSC-0910 and MCA-0965/TSC-0910) met the standards of good clinical practice and were approved by the institutional review board of the Rockefeller University. Participant cohort information is described in Table S1 and in a previous publication (Weymar et al., 2022).

### CRISPR RNA (crRNA) design

crRNAs were designed using the Integrated DNA Technologies crRNA design tool (http://www.idtdna.com) and synthesized by Integrated DNA Technologies as Alt-R CRISPR/Cas9 RNAs. crRNA sequences are listed in Table S2.

### Homology-directed repair template (HDRT) preparation and AAV production

HDRT sequences (Table S1) were cloned into a pAAV vector using the Gibson assembly technique (Gibson et al., 2009) with the NEBuilder HiFi DNA Assembly Master Mix (cat. E2621L; New England Biolabs). AAVs carrying each HDRT were produced as previously described (Hartweger et al., 2023). Our strategy is optimized for targeting efficiency and would not accommodate a full-length provirus that cannot be incorporated into AAV.

### Nucleofection of Jurkat and primary human CD4^+^ T cells

Genome editing was achieved through a previously described combination of CRISPR targeting and AAV-mediated homologous recombination (Martin et al., 2019).

Primary CD4^+^ T cells were enriched from healthy donor cryopreserved PBMCs by negative selection (CD4^+^ T Cell Isolation Kit human, cat. 130-096-533; Miltenyi Biotec) and activated for 72 h with 10 µg/ml of plate-bound Ultra-LEAF purified anti-human CD3 monoclonal antibody (clone OKT3, cat. 317325; BioLegend) and 5 µg/ml of soluble Ultra-LEAF purified anti-human CD28 monoclonal antibody (clone 28.2, cat. 302933; BioLegend) in R10 medium (RPMI-1640 medium supplemented with 10% heat-inactivated FBS, 10 mM Hepes, antibiotic-antimycotic (1×) and 2 mM *L*-glutamine [all from Gibco]) supplemented with recombinant human IL-2 (50 U/ml; cat. 10799068001; Roche).

For a 100 µl transfection, 1 µl of 200 µM of gene-specific crRNA and 1 µl 200 µM trans-activating crRNA (tracrRNA) in duplex buffer (all from Integrated DNA Technologies) were combined, denatured at 95°C for 5 min, and renatured for 5 min at room temperature. Then, 5.6 µl PBS and 2.4 µl 61 µM Cas9 (Alt-R *S.p.* Cas9 Nuclease V3, cat. 1081058; Integrated DNA Technologies) were added and the mixture was incubated for 20 min. Finally, 4 µl 100 µM electroporation enhancer in duplex buffer was added to 10 µl of the RNPs and the mixture was incubated for a further 1–2 min.

Nucleofection of Jurkat cells was performed using SE Cell Line 4D-Nucleofector X Kit L (cat. V4XC-1012; Lonza), while the P3 Primary Cell 4D-Nucleofector X Kit L (cat. V4XP-3024; Lonza) was used for primary T CD4^+^ T cells, as previously described (Hultquist et al., 2019).

After nucleofection cells were transferred into a 12-well plate containing R10 medium and the corresponding HDRT-carrying AAV.

### Jurkat and primary human CD4^+^ T cells

3 days after nucleofection, cells were stained with Fixable Viability Dye eFluor 780 (cat. 65-0865-14; Invitrogen) and single-cell sorted using a FACSymphony S6 using FACSDiva software (version 9.5.1; BD Biosciences) into R10 (Jurkats) or activation medium (R10 + 50 U/ml IL-2, 0.1 µg/ml of anti-CD3 and -CD28 monoclonal antibodies) plus feeder cells (1 × 10^6^/ml NK and CD8^+^ T cell–depleted, 5,000 rad irradiated allogeneic PBMCs). 3 weeks after sorting, a small fraction of the cells was collected, genomic DNA was extracted (QuickExtract DNA Extraction Solution, cat. QE09050; Lucigen), and the remainder was restimulated with fresh medium and cultured for an additional 14 days (Jones et al., 2016).

Clones were screened for integration with combinations of HDRT- and gene-specific primers, covering the entire construct and the integration sites (Table S2). PCRs were performed using Phusion Green Hot Start II High-Fidelity DNA Polymerase (cat. F537S; Thermo Fisher Scientific) and Sanger sequencing of gel extracted amplicons performed (Azenta Life Sciences).

### CD4^+^ T cells from individuals on ART

CD4^+^ T cells harboring intact latent HIV-1 proviruses were enriched using their TCRs (Weymar et al., 2022).

CD4^+^ T cells from participants 603 (Rockefeller University IRB–approved Protocols MCA-0866/TSC-0910) and 5104 (Protocols MCA-0965/TSC-0910) were isolated by magnetic separation using CD4^+^ T Cell Isolation Kit, human (cat. 130-091-155; Miltenyi Biotec) and CD45RA MicroBeads, human (cat. 130-045-901; Miltenyi Biotec), and prepared for cell sorting exactly as described (Weymar et al., 2022). Cells from 603 (CD4^+^TRBC1^+^TRBV19^+^) and 5104 (CD3^+^CD4^+^TRBC1^+^TRBV^−^) were sorted into 96 well “U” bottom plates (five cells per well) containing 200 µl of activation media (composition described above), plus feeder cells, supplemented with the following ARVs: 1 µM tenofovir, 1 µM emtricitabine, 1 μM nevirapine, and 10 µM T-20. Cell sorting was performed on FACSymphony S6 using FACSDiva software (version 9.5.1; BD Biosciences) and data were analyzed using FlowJo (version 10.10.0; BD Biosciences). The culture media was replaced twice a week to replenish ARVs and avoid new infections.

3 weeks after sorting, cells were screened for the presence of the HIV clone of interest by Env PCR and sequencing (Salazar-Gonzalez et al., 2008). Cell lines containing clones of interest were expanded in a T25 culture flask in 10 ml of activation medium. The frequency of latent cells in the cultures was determined 3 weeks after expansion by sorting single cells into 10 µl of RLT buffer (cat 79216; Qiagen) using Agencourt RNAClean XP magnetic beads (cat. A63987; Beckman Coulter) for DNA preparation and Env PCR.

### Activation of cultured cells

Jurkat cells were activated in R10 medium supplemented with PMA (25 ng/ml, cat. P1585; Sigma-Aldrich) and ionomycin (1 μg/ml, cat. I9657; Sigma-Aldrich) or R10 supplemented with anti-human CD3 and CD28 antibodies (as above) for 24 h.

Primary CD4^+^ T cells carrying reporter constructs or those obtained from individuals 603 and 5,104 or controls, 3–4 weeks after the last stimulation in activation medium plus feeder cells, were stimulated with activation medium (described above) for either 24 h or 1 week. In parallel, cells were maintained in resting conditions by culturing in simple R10 or R10 supplemented with 10 ng/ml of recombinant human interleukin-7 (IL-7, cat. BT-007-010; R&D Systems) for 1 wk. For LRA experiments, resting cells were cultured for 24 h in R10 containing (+)-JQ1 (1 µM, cat. SML1524-5MG; Sigma-Aldrich), prostratin (2.5 µM, cat. P0077-1MG; Sigma-Aldrich), SAHA (0.5 µM, cat. SML0061-5MG; Sigma-Aldrich), romidepsin (5 nM, cat. 14083; Active Motif), or panobinostat (50 nM, cat. SML3060-10MG; Sigma-Aldrich).

### Flow cytometric analysis

T cell clones were assessed for GFP expression by flow cytometry. Cells were stained with Fixable Viability Dye eFluor 780 and the following monoclonal antibodies: TexasRed-PE conjugated anti-human CD3 (clone 7D6, cat. MHCD0317; Invitrogen); PerCP/Cyanine5.5 conjugated anti-human CD4 (clone OKT4, cat. 317428; BioLegend); and Brilliant Violet–conjugated anti-human CD25 (clone BC56, cat. 302634; BioLegend).

HIV Gag p24 expression was evaluated by flow cytometry in CD4^+^ T cells derived from participants 603 and 5104. Cells were fixed and permeabilized using BD Cytofix/Cytoperm Fixation/Permeabilization Kit (cat. 554714; BD Biosciences) and stained for Gag p24 protein with anti-HIV-1 core antigen monoclonal antibody (clone KC57, cat. 6604665; Beckman Coulter).

Cells were analyzed in a FACS Symphony A5 flow cytometer running FACS Diva software (version 8.5; BD Biosciences) and data were analyzed using FlowJo (version 10.10.0; BD Biosciences). Only live cells, CD25^high^ (activation conditions) or CD25^low^ (resting conditions) were considered for measuring GFP or p24 fluorescence.

### qPCR

RNA was purified from the bulk clonal populations using the RNeasy Plus Micro kit (cat. 74034; Qiagen) and converted to cDNA using SuperScript III Reverse Transcriptase (cat. 18080-093; Invitrogen) using a combination of random primers (cat. 48190011; Invitrogen) and LTR-specific primers for reporter primary cells, plus primers to polyA, nef, tat-rev, and pol for HIV^+^ cells (Table S2) (Einkauf et al., 2022).

qPCR was performed on cDNA using TaqMan Fast Advanced Master Mix for qPCR (cat. 4444558; Applied Biosystems) with 500 nM primers and 250 nM probe plus 1 µl of cDNA. For relative quantification of expression, normalization was performed against the *PPIA* gene. Primers and probes used for quantification of LTR, eGFP, and nanoLuciferase were from Integrated DNA Technologies (Table S2). For host gene expression, we used predesigned PrimeTime qPCR Assays (Integrated DNA Technologies): *ZNF407* – Hs.PT.58.4027445; *ZNF140* – Hs.PT.58.39945645; *KDM2A* – Hs.PT.58.815648; *ZNF460* – Hs.PT.58.24604991; *KCNA3* – Hs.PT.58.2591067.g; *ATP2B4* – Hs.PT.56a.26489531.g; *PPIA* – Hs.PT.58v.38887593.g; PIKFYVE – Hs.PT.58.26959495 and Hs.PT.58.40848161; and FIZ1 – Hs.PT.58.2780215 and Hs.PT.58.5062508. HIV-specific primers for poly-A, nef, tat-rev, pol, and long LTR were as previously described (Palmer et al., 2003; Gaebler et al., 2019; Einkauf et al., 2022) and provided in Table S2. Relative expression was calculated as $2^{-\left(Ct_{target}-Ct_{ref}\right)}$. The number of LTR transcripts were enumerated in a qPCR reaction using the same LTR primers as above. A standard curve of 10-fold serial dilutions of a plasmid carrying a LTR sequence was created by plotting known DNA copy numbers in each dilution to their respective Ct value. Ct values measured for LTR in each sample were input in the standard curve and the transcript copy number thus obtained was divided by the number of cells, or infected cells for HIV^+^ cell cultures, equivalent to input volume of cDNA in the reaction to obtain the number of LTR transcripts per cell.

### ATAC-seq

Dead cells were removed from the culture using the Dead Cell Removal Kit (cat. 130-090-101; Miltenyi Biotec). Then, 100,000 Jurkat cells or primary CD4^+^ T cells were used per reaction. For primary T cells, each clone was sampled in both resting and activated conditions as described above. Three replicates were performed per condition. Cells were lysed and DNA tagmented, purified, and amplified using an ATAC-seq kit (cat. 53150; Active Motif). The libraries were sequenced using Illumina NextSeq 550 paired-end sequencing.

The human genome assembly hg38 was modified adding HIV inserts at specified loci using custom scripts. Subsequently, all libraries were mapped to this modified genome using bowtie2 with the parameters --local --very-sensitive-local. Duplicated reads were then removed with Picard MarkDuplicates with default parameters. Regions with enriched signal throughout the genome were obtained using macs2 for peak calling with the parameters --format BAMPE --nomodel –nolambda --cutoff-analysis, and genome blacklist regions (https://github.com/Boyle-Lab/Blacklist) were removed (Amemiya et al., 2019). Differentially accessible regions were identified using edgeR.

### 10X Genomics

10X Genomics gene expression and V(D)J libraries were generated with the Chromium Single Cell 5′ Library & Gel Bead Kit (cat. PN-1000014; 10X Genomics) and Chromium Single Cell V(D)J Enrichment Kit, Human T Cell (cat. PN-1000005; 10X Genomics) as described in the 10X Genomics protocol. The 5′ expression library and the V(D)J library were sequenced with NovaSeq 6000 S1 (100 cycles) (cat. 20012865; Illumina).

### Single-cell RNA-seq (scRNA-seq) and single-cell TCR-seq processing

scRNA-seq binary base call (BCL) files underwent demultiplexing and were transformed into FASTQ files using BCLtoFastq, followed by alignment against a modified hg38 that includes specific HIV sequences for each subject utilizing CellRanger (v7.2.0). Analysis was performed in R studio using Seurat (v5.0.1). Cells exhibiting mitochondrial content over 10% and/or feature counts outside the 200 to 2,500 range were excluded. Sample batches were merged, then normalized, and scaled using SCTransform. Single-cell TCR-seq FASTQ files were aligned to the standard CellRanger VDJ reference using CellRanger (v7.2.0). The resulting contig annotations were filtered and examined in R studio. Cells harboring TCRs identical to those found by Weymar and colleagues (Weymar et al., 2022) were classified as latent cells, whereas those with diverse TCRs were identified as non-infected cells.

### Mapping scRNA-seq from cultured cells to previously published uniform manifold approximation and projection (UMAP)

The UMAP from a previous report (Weymar et al., 2022) served as the reference, and the cultured cells from each subject were anchored and mapped using the FindTransferAnchors and MapQuery functions from Seurat (reference.reduction = "pca", dims = 1:30, reduction.model = umap, refdata = clusters). Only cells with a prediction score of 0.95 or higher for label transferring were selected to create Fig. 5 B.

### Statistical analysis

Pearson’s correlation coefficients, r, and two-tailed P values were computed for the relationship between the host gene and reporter LTR expression. Statistical analyses were performed in GraphPad PRISM version 10.

Analysis of ATAC-seq and single-cell sequencing data were processed and analyzed using R studio version 2023.12.1+402 running R version 3.3.0.

### Online supplemental material

Fig. S1 shows quantification of flow cytometry data and LRA experimental results. Fig. S2 shows the levels of reporter LTR transcription at two additional integration sites. Figs. S3 and S4 show chromatin accessibility around the reporter construct integration site in Jurkat and primary CD4^+^ T cell clones, respectively. Fig. S5 shows HIV-1 RNA splice variants mapped to the genome from 10X Genomics data. Table S1 details the clinical characteristics of the participants of previous studies whose cells were used in the current work. Table S2 lists crRNA sequences, annotated HDRT sequences, and primer sequences for the various assays.

## Supplementary Material

Table S1 shows the clinical characteristics of the study participants.

Table S2 lists crRNA sequences, annotated HDRT sequences, and primer sequences for the various assays.

## Data Availability

10X Genomics (underlying Figs. 5 and S5) and ATAC-seq data (underlying Figs. 4, S3, and S4) generated in this project are publicly available as of the date of publication and are archived in the National Center for Biotechnology Information (NCBI) Gene Expression Omnibus (GEO) database under accession number GSE268010. The data partially used in Fig. 5 are openly available at NCBI GEO accession number GSE204756. All codes used in this work are publicly available as of the date of publication. The code for single-cell analysis is available at https://doi.org/10.5281/zenodo.6950427. The code to identify the TCR of the latent clone of interest is publicly available on GitHub (https://github.com/victor-ramos/demultiplex_and_assembly_TCR) and Zenodo (https://doi.org/10.5281/zenodo.6954076).
