# Correction: Transcription of HIV-1 at sites of intact latent provirus integration

**DOI:** 10.1084/jem.2024039105162025c

**Published:** 2025-05-23

**Authors:** Ana Rafaela Teixeira, Cintia Bittar, Gabriela S. Silva Santos, Thiago Y. Oliveira, Amy S. Huang, Noemi Linden, Isabella A.T.M. Ferreira, Tetyana Murdza, Frauke Muecksch, R. Brad Jones, Marina Caskey, Mila Jankovic, Michel C. Nussenzweig

Vol. 221, No. 9 | https://doi.org/10.1084/jem.20240391 | August 14, 2024

Upon further analysis after publication, the authors found that the HIV integration site for clone 5104 is in the satellite region of chromosome 22 (chr22:10,229,326-10,229,469) and not in the intron of the *ATP2B4* gene as stated in the original version of the article. The main conclusions of the article remain unchanged.

Changes have been made in the "Intact latent HIV-1 proviruses" section of the Results, the third and sixth paragraphs of the Discussion, Fig. 5 G, and the Fig. 5 legend. The corrected text, original and corrected figures, and figure legend are shown here, with added text in bold and underlined. The errors appear in print and in PDFs downloaded before May 16, 2025.

## Results

### Intact latent HIV-1 proviruses

CD4^+^ T cells carrying intact latent HIV-1 proviruses are rare and express no singular cell surface marker that distinguishes them from T cells other than their unique T cell receptor (TCR) (Cohn et al., 2018; Collora et al., 2022; Einkauf et al., 2022; Weymar et al., 2022; Sun et al., 2023; Wu et al., 2023). To examine HIV-1 expression in authentic latent reservoir cells, we enriched CD4^+^ T cells carrying replication-competent latent proviruses by means of their specific TCRs (Weymar et al., 2022)**:****(**#603 and #5104 with HIV-1 integrated into *ZNF486* and ***ATP2B4*)human satellite 3 (HSat3) DNA in Chr22**. The sorted cells were then expanded under limiting dilution conditions in the presence of irradiated feeder cells and antiretroviral drugs (ARVs). The presence and frequencies of the infected clones in the expanded cells lines were verified by PCR assays for their specific TCRs and HIV-1 sequences (Fig. 5 A). Cell lines were obtained in which at least half of the cells represented the T cell clones of interest with intact proviruses integrated into *ZNF486* and ***ATP2B4* genesChr22 HSat3**.

Primary CD4^+^ T cells carrying intact latent proviruses isolated directly from individuals 5104 and 603 are enriched in T cell populations with specific transcriptional profiles (Weymar et al., 2022). To determine the transcriptional profile of cultured lines of CD4^+^ T cells carrying integrated HIV-1 proviruses in *ZNF486* and ***ATP2B4* genesChr22 HSat3**, we performed single-cell mRNA sequencing experiments using the 10X Genomics platform. Specific TCR expression was used to identify the latent cells and map them onto our previous data set using a cut-off of 95% confidence (Fig. 5, B and C and Materials and methods) (Weymar et al., 2022). Cultured and ex vivo cells containing latent HIV-1 proviruses showed closely related transcriptional profiles (Fig. 5, B and C).

The enriched populations of resting (3 wk after activation) and activated (24 h after anti-CD3 and -CD28 monoclonal antibody stimulation) CD4^+^ T cells carrying the 5104 and 603 HIV-1 proviruses were initially examined for p24 expression by flow cytometry (Fig. 5 D). **Like the reporters in Jurkat and CD4**^**+**^**cell lines, theThe** HIV-1 provirus integrated into ***ATP2B4*, an actively transcribed gene, Chr22 HSat3** showed expression under both resting and activated conditions (Fig. 5 D). In contrast, the provirus integrated into ZNF486, a gene expressed at only low levels, failed to show detectable p24 expression under resting or activated conditions (Fig. 5 D). 

HIV-1 LTR, *gag*, and *env* mRNA expression were measured by qPCR corrected for the fraction of infected cells in the culture (Fig. 5 E and Materials and methods). The HIV-1 provirus integrated into ***ATP2B4*Chr22 HSat3** showed similar levels of expression of LTR, *gag*, and *env* under resting and activated conditions. In addition, we found multiple spliced transcripts associated with productive infection in single-cell transcriptome analysis (Fig. S5). In contrast, the HIV-1 provirus integrated into *ZNF486* only showed LTR, gag, and env transcripts after activation (Fig. 5 E). When compared to productive infection with HIV-1_YU2_, the proviruses in ***ATP2B4*Chr22HSat3** and *ZNF486* were expressed at >100 and >27,000-fold lower levels, respectively (Fig. 5 F). When compared with reporter proviruses integrated into *ATP2B4*, the level of LTR expression obtained from cultured CD4^+^ T cells carrying latent HIV-1 was 10- and 100-fold higher than in primary CD4^+^ T cells and Jurkat cells, respectively. We conclude that authentic latent HIV-1 proviruses integrated at different sites in the genome of CD4^+^ T cells show different levels of expression and ability to respond to activation. In addition, proviruses integrated at latent sites are expressed at far lower levels than in productive infection.

To determine whether the latent HIV-1 proviruses in the cultured cells altered neighboring host gene transcription (***ATP2B4* and***ZNF486*), their expression was compared with non-infected cells from the same culture in the 10X Genomics data set. We found no significant difference in the expression of **genesthe gene** neighboring the integrated HIV-1 provirus between infected and non-infected cells irrespective of activation (Fig. 5 G).

**Discussion** (*third paragraph*)

We found little effect on transcription of neighboring genes by reporter proviruses or HIV-1 integrated at **eightnine** sites of intact latent proviral integration in Jurkat cell lines, primary CD4^+^ T cells, or CD4^+^ T cells obtained from PLWH and grown in culture. Initially, intact latent proviruses are preferentially found in introns of genic regions in the opposite transcriptional orientation to the transcriptional start site (Einkauf et al., 2019; Huang et al., 2021). Over time, in chronically infected individuals under ART and elite controllers, intact latent proviruses accumulate in ZNF genes and non-genic regions (Einkauf et al., 2019; Jiang et al., 2020; Huang et al., 2021). Our collection of integration sites mirrors this selection, and therefore the observation that intact latent proviruses have little measurable effect on the neighboring genome may be biased by the origins of the sample set. Nevertheless, the integrations examined include unique proviruses found only once and others found in expanded clones. 

(*sixth paragraph*)

We studied two cell lines derived directly from latently infected cells obtained from PLWH. Proviral transcription was detected in cells where HIV-1 was integrated in **a highly expressed gene, *ATP2B4*,Chr22 HSat3**, but not in cells carrying a provirus integrated in *ZNF486*, a gene expressed at low levels. **Although limited to two examples, the results are congruent with our observations using indicator proviruses in Jurkat and primary CD4**^**+**^**T cells.** In all cases, the levels of HIV-1 transcription from sites of intact latent integration were at least two orders of magnitude below the levels found in productively infected cells. Although insufficient to kill all the infected cells, the level of proviral transcription from the ***ATP2B4*Chr22 HSat3** integration was sufficient to produce an infectious virus (data not shown). Thus, latent CD4^+^ T cells carrying an HIV-1 provirus integrated into ***ATP2B4*Chr22 HSat3** are also likely to produce enough viral protein to be visible to the immune system.

**Figure:**
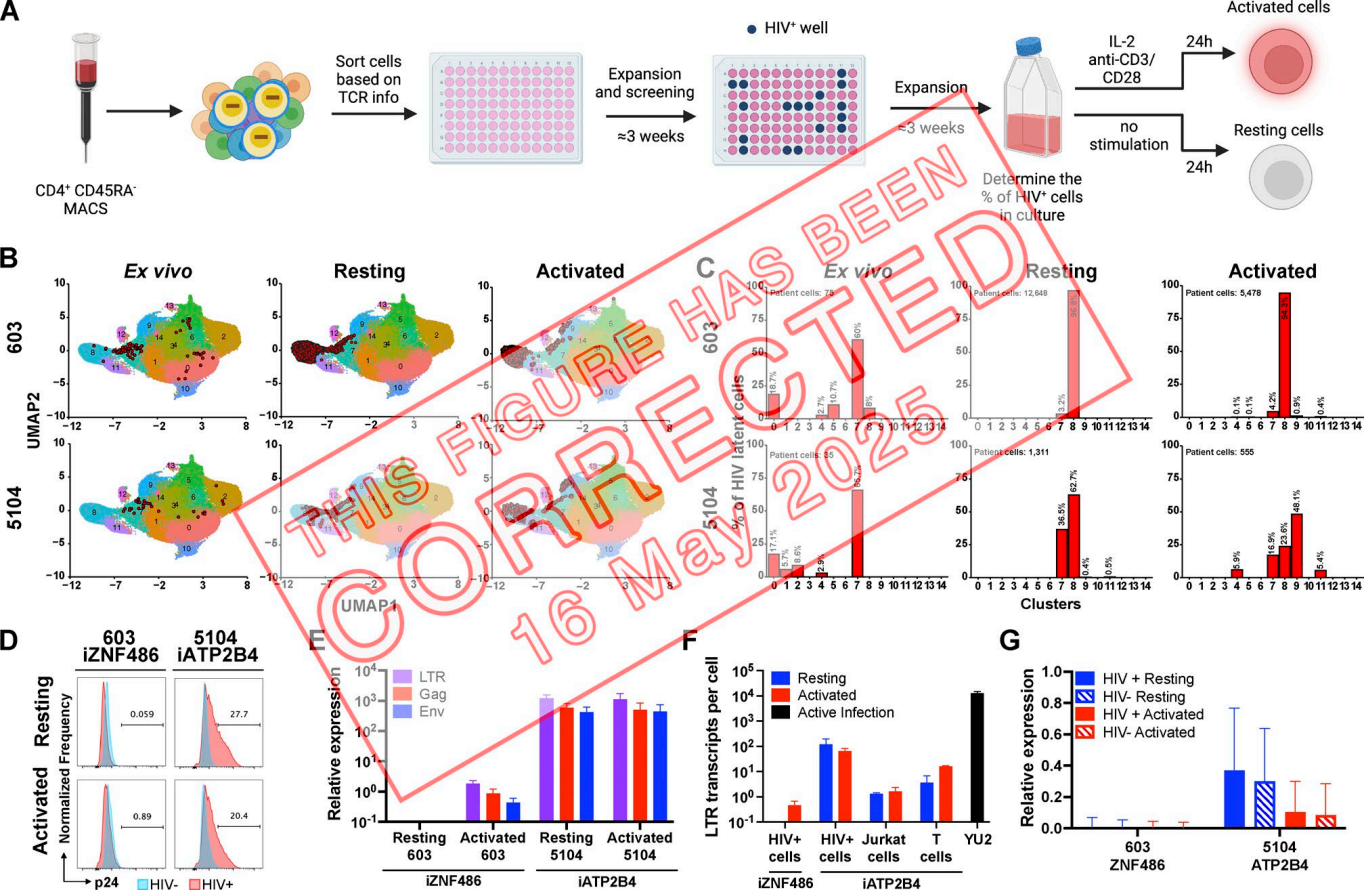


**Figure 5.**
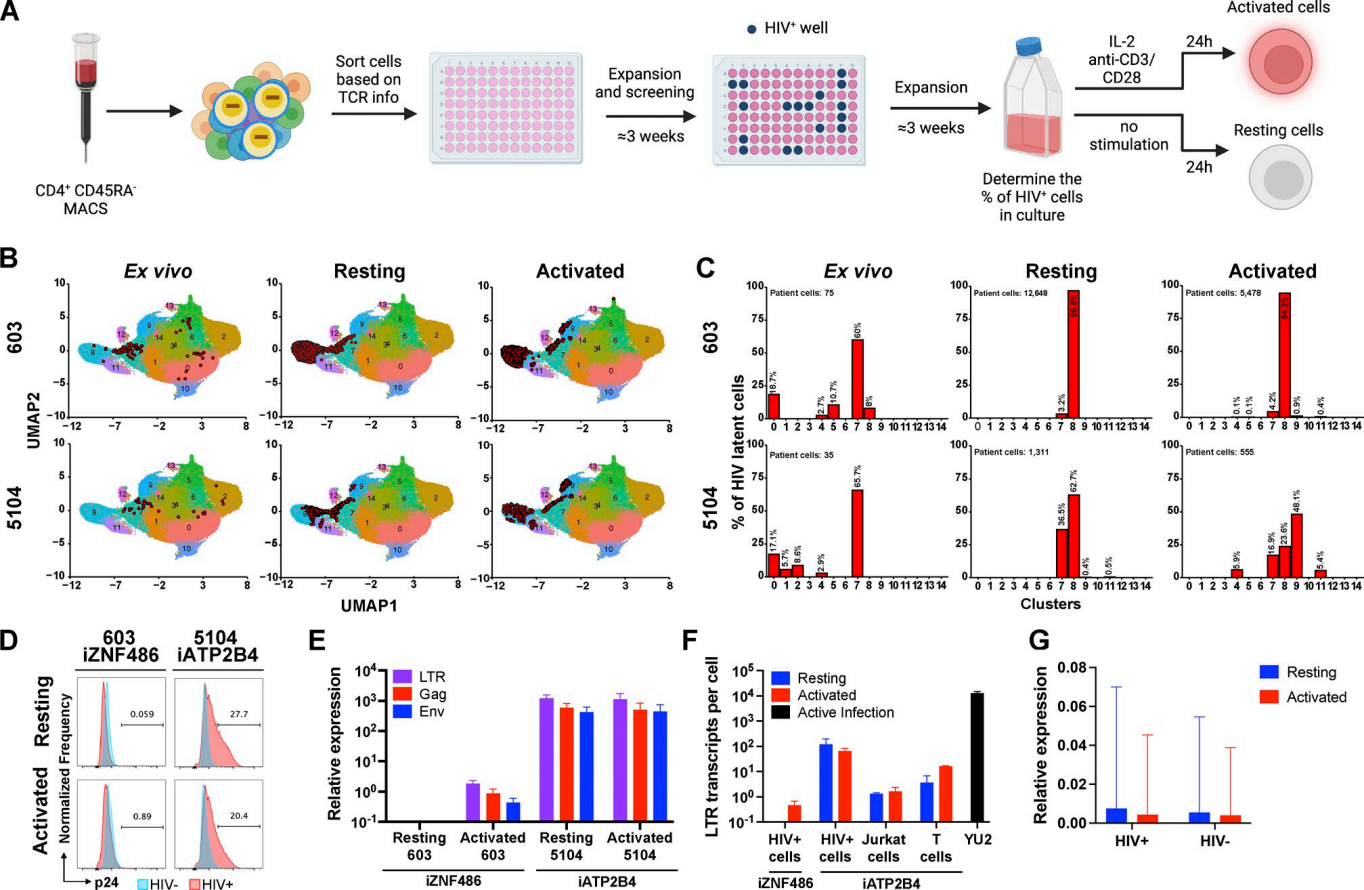
**HIV-infected cells from PLWH. (A)** Schematic representation of the methods used to grow out HIV-1–infected cells from ART-suppressed individuals (Weymar et al., 2022). Created with https://BioRender.com. **(B and C)** UMAP of 10X single-cell gene expression data showing the position of the cells expressing the latent clones’ specific TCR (red dots) (B) and the fraction of latent cells in each cluster (C), for participants 603 (upper panels) and 5104 (lower panels) from ex vivo cells (left panels, data from Weymar et al. [2022]), and cultured cells under resting (middle panels) and activated (right panels) conditions. **(D)** Histograms show HIV-1 Gag p24 expression in HIV^+^ cells (red) and non-infected cells (blue) of cultures derived from 603 to 5104, under resting (upper panel) and activated (lower panel) conditions. **(E)** Relative expression of LTR (purple bars), *gag* (red bars), and *env* (blue bars) by qPCR for 603 and 5104 HIV^+^ cells under resting and activated conditions. Bars represent the mean relative expression of two independent assays (biological replicates) ± SD. **(F)** LTR transcripts per cell determined by qPCR, in cells from 603 to 5104 and in Jurkat and primary T cells reporter lines with proviruses integrated into *ATP2B4*, under resting (blue) and activated (red) conditions and HIV-1_YU2_ controls. Bars represent the mean of two independent experiments (biological replicates) ± SD. **(G)** Relative expression determined by 10X Genomics single-cell mRNA sequencing of host **genesgene** neighboring HIV-1 proviral integration (*ZNF486***, participant 603 and *ATP2B4*, participant 5104**) in HIV-infected (HIV^+^**, full bars**) and non-infected (HIV^−^**, striped bars**) cells from the same cell population under resting (blue bars) and activated (red bars) conditions. Bars represent the mean of the respective population from one assay ± standard deviation representing population variance.

