# Supplementary material for: Transcription of HIV-1 at sites of intact latent provirus integration
Source: J Exp Med. 2024 Aug 14;221(9):e20240391. doi: 10.1084/jem.20240391 (PMC11323366; doi:10.1084/jem.20240391)
Supplement: Table S1 — shows the clinical characteristics of the study participants. [file jem_20240391_tables1.docx]

**Table S1. Clinical characteristics**

| **ID** | **Age** | **Sex** | **Race** | **Year**  **HIV-1 Dx** | **Year ART initiation** | **Uninterr. ART**  **(years)** | **Viral Load (copies/ml)** | **CD4^+^ T cell count** | **Reported nadir** | **ART Regimen** |
| --- | --- | --- | --- | --- | --- | --- | --- | --- | --- | --- |
| **603** | **43** | **Male** | **White/**  **Hispanic** | **12** | **10** | **10** | **<20** | **300** | **693** | **EFV/TDF/FTC** |
| **5104** | **35** | **Male** | **Black** | **7** | **7** | **7** | **<20** | **450** | **1,006** | **DTG/TDF/FTC** |

EFV, Efavirenz; TDF: tenofovir disoproxil fumarate; FTC: emtricitabine; DTG: dolutegravir.
